# Liana Abundance, Diversity, and Distribution on Barro Colorado Island, Panama

**DOI:** 10.1371/journal.pone.0052114

**Published:** 2012-12-21

**Authors:** Stefan A. Schnitzer, Scott A. Mangan, James W. Dalling, Claire A. Baldeck, Stephen P. Hubbell, Alicia Ledo, Helene Muller-Landau, Michael F. Tobin, Salomon Aguilar, David Brassfield, Andres Hernandez, Suzanne Lao, Rolando Perez, Oldemar Valdes, Suzanne Rutishauser Yorke

**Affiliations:** 1 Department of Biological Sciences, University of Wisconsin-Milwaukee, Milwaukee, Wisconsin, United States of America; 2 Smithsonian Tropical Research Institute, Balboa, Republic of Panama; 3 Department of Plant Biology, University of Illinois, Urbana, Illinois, United States of America; 4 Program in Ecology, Evolution, and Conservation Biology, University of Illinois, Urbana, Illinois, United States of America; 5 Department of Ecology and Evolutionary Biology, University of California Los Angeles, Los Angeles, California, United States of America; 6 ETSI Montes, Universidad Politécnica de Madrid, Madrid, Spain; Odum School of Ecology, University of Georgia, United States of America

## Abstract

Lianas are a key component of tropical forests; however, most surveys are too small to accurately quantify liana community composition, diversity, abundance, and spatial distribution – critical components for measuring the contribution of lianas to forest processes. In 2007, we tagged, mapped, measured the diameter, and identified all lianas ≥1 cm rooted in a 50-ha plot on Barro Colorado Island, Panama (BCI). We calculated liana density, basal area, and species richness for both independently rooted lianas and all rooted liana stems (genets plus clones). We compared spatial aggregation patterns of liana and tree species, and among liana species that varied in the amount of clonal reproduction. We also tested whether liana and tree densities have increased on BCI compared to surveys conducted 30-years earlier. This study represents the most comprehensive spatially contiguous sampling of lianas ever conducted and, over the 50 ha area, we found 67,447 rooted liana stems comprising 162 species. Rooted lianas composed nearly 25% of the woody stems (trees and lianas), 35% of woody species richness, and 3% of woody basal area. Lianas were spatially aggregated within the 50-ha plot and the liana species with the highest proportion of clonal stems more spatially aggregated than the least clonal species, possibly indicating clonal stem recruitment following canopy disturbance. Over the past 30 years, liana density increased by 75% for stems ≥1 cm diameter and nearly 140% for stems ≥5 cm diameter, while tree density on BCI decreased 11.5%; a finding consistent with other neotropical forests. Our data confirm that lianas contribute substantially to tropical forest stem density and diversity, they have highly clumped distributions that appear to be driven by clonal stem recruitment into treefall gaps, and they are increasing relative to trees, thus indicating that lianas will play a greater role in the future dynamics of BCI and other neotropical forests.

## Introduction

Lianas (woody vines) are a common plant growth-form in lowland tropical forests where they affect many aspects of tropical forest dynamics and function. Lianas reduce tropical tree recruitment, growth, survival, fecundity, and diversity [1]–[10]. At the community level, lianas appear to influence tree species composition by competing intensely with certain tree species, but not with others [9], [11]–[13]. At the ecosystem level, lianas have the potential to substantially alter forest carbon, nutrient, and water dynamics by decreasing whole-forest carbon sequestration and storage, redistributing nutrients horizontally across the forest landscape, and reducing available soil moisture during seasonal droughts [14]–[16].

Determining landscape-level distributions and spatial patterns of lianas is essential to accurately predict the forest areas and processes on which lianas will have the greatest influence, to assess the community ecology of lianas, and to provide insights into the mechanisms that control liana species abundance and distribution within forests. For example, a highly aggregated liana distribution would be consistent with the hypothesis that within-forest liana distribution is driven by treefall gaps [8], [17], and thus lianas would likely have a large effect on gap-phase regeneration, e.g. [9]. Many liana species colonize gaps primarily via stems that fall with the gap-making tree and then rapidly propagate through clonal reproduction [1], [3], [18], [19]. Thus, we would expect the liana species that can capitalize on treefall gaps should arrive soon after gap formation, possibly via clonal reproduction, and that most of the stems within aggregations would be similarly sized. In contrast, liana species that recruit into the understory (from either seed or by clonal stems) should colonize more continuously over time, regardless of canopy openness, and thus we would expect to find much more variation in stem sizes within aggregations.

Currently, there exists little information on landscape-level liana distribution and spatial structure, and how the production of clonal stems influences these factors. Most studies of liana ecology are conducted at relatively small spatial scales (<1 ha; e.g. [1], [2], [11], [20]) or include only very large lianas (≥100 mm diameter; e.g. [21], [22]). Thus, previous studies rarely provide a sample size large enough to examine how lianas are distributed across the landscape. Few studies have systematically examined the prevalence of clonality of many liana species, and how this mode of regeneration can dictate liana abundance and distribution within a forest (but see [23]). Furthermore, few studies have compared liana and tree spatial distributions, which may differ substantially if the distribution of each growth form is determined by different mechanisms. For example, while the distribution of many tree species within a forest may be influenced by edaphic factors, e.g. [24], [25], within-forest liana distribution may be more strongly influenced by the occurrence of canopy gaps [8], [17], [26].

Moreover, the relative abundance and biomass of lianas appear to be increasing throughout the neotropics [27]. On Barro Colorado Island (BCI), liana productivity and flower production has increased substantially compared to trees [28], [29], and the proportion of trees infested by lianas has increased from 32% to nearly 75% over the past four decades [10]. In 1979, lianas were censused in ten 0.1 ha plots on the central plateau on BCI [1], in the area where the BCI 50-ha plot is now located, but until now we lacked comparable data to examine whether liana density has increased on BCI. An increase in liana density is likely to have significant consequences for community and ecosystem level processes in tropical forests [30].

In this study, we present the most comprehensive spatially contiguous sampling of lianas ever conducted and we provide the first full description of the liana community of the BCI 50-ha plot. We quantified the abundance, diversity, and distribution of all lianas (≥1 cm diameter) rooted in the BCI 50-ha forest dynamics plot, located in central Panama. We used this dataset to address the following five questions.

1. What is the contribution of lianas to woody species richness, stem density, and basal area in the BCI 50-ha plot?
2. What is the spatial structure and distribution of lianas in the BCI 50-ha plot and how do distributions vary among liana species?
3. How do liana species vary in their production of clonal stems, and do liana species with a high propensity for clonal reproduction have higher stem density and larger mean stem diameter than species with a low propensity for clonal reproduction?
4. How do spatial aggregation patterns of liana species compare to those of trees? Furthermore, how do spatial aggregation patterns of liana species with many clonal stems compare to liana species with few clonal stems?
5. Has liana density (both absolute and relative to trees) increased in the BCI forest over the past 30 years?

## Methods

### Study Site

The forest of the BCI 50-ha plot is almost entirely old-growth seasonally moist lowland tropical forest, with a small (∼1 ha) portion of late secondary forest (>100 yrs-old [31]). Mean annual rainfall is around 2600 mm, with a dry season from December until May. In 1980–1982, all trees ≥1 cm diameter were measured, mapped, identified, and tagged in the 50 ha plot and have been censused every five years thereafter [32]. Tree data used in this study are from the 2005 census (published in 2011 on the CTFS website: https://ctfs.arnarb.harvard.edu/webatlas/datasets/bci/abundance/). Because this study was conducted on the Barro Colorado Nature Monument, no specific permits were required. Descriptions of the geology, climate, flora and fauna of BCI, as well as the census of the 50-ha plot can be found in references [32]–[35].

### Liana Census of the BCI 50-ha Plot

From February through December 2007, we tagged, mapped, measured, and identified all rooted lianas ≥1 cm diameter using the census methods described in [35] and [36]. We measured liana stem diameter 1.3 m from the rooting point and tied a uniquely numbered aluminum tag to the stem. We mapped the rooting point of each liana using the existing 20,000 5×5 m grid markers to aid in recording the precise location of the stem (within 0.5 m), and we digitized all maps. We considered each separately rooted liana that was not connected aboveground to any other liana in the study to be an “apparent genet” because it appeared to be a genetically distinct individual [5]. When a single liana had multiple rooted stems ≥1 cm in diameter, we considered the largest diameter stem to be the “principal stem” (and apparent genet), and each of the smaller stems to be the clonally produced ramets [5], [35], [36]. Thus, throughout the manuscript we use the terms “principal stem” and “clone” to distinguish between these two stem types. Stems that branched within 40 cm of the rooting point are notoriously difficult to measure accurately [35]; therefore, for these stems, we measured all branches 1.3 m from the rooting point and calculated basal area as the sum of the branch basal areas (follows [5], [35], [36]). Branches were not considered to be clones because they did not have a separate root system and were analogous to the branches of a tree rather than a clonal stem [5], [35], [36].

We identified lianas to species in the field using a combination of stem, leaf, and flower characteristics, and we were able to identify 98.5% (46,495) of the individuals to species. We were unable to identify to species 1.5% (688) of the individuals, usually because we were unable to see the leaves to confirm the species identity. We were also unable to identify to species 27 individuals in the genus *Smilax* (Smilacaceae), so we excluded this genus, along with the unidentified stems, from all species-level analyses.

### Quality Control

During the course of the 10-month census, we reduced errors by implementing five levels of quality control [35]. **1)** All datasheets were examined weekly for anomalies or missing data and anomalous data were checked and revised in the field. **2)** Two field supervisors used the maps to locate lianas in each of the 1250 20×20 m quadrats to ensure that the liana locations were mapped correctly. Mapping errors were corrected in the field prior to digitizing the maps. **3)** The field supervisors checked stem measurement locations for randomly selected lianas in each 20×20 m quadrat to ensure that the correct stem measurement protocol was followed; errors were corrected in the field. **4)** The field supervisors checked the accuracy of the diameter measurements by re-measuring approximately 5% of the lianas in each 20×20 m quadrat, thus ensuring that there were no systematic measurement errors among the field technicians. **5)** We quantified the error rate for our estimate of liana clonal reproduction by randomly selecting and revisiting 5% (64) of the 1250 20×20 m quadrats and counting and re-measuring all rooted liana ramets (clones) that were ≥1 cm diameter and still connected to the principal stem (3,768 stems total). The mean error rate for determining clonal reproduction was <1% at both the quadrat level (0.89%) and at the individual liana level (0.50%).

### Diversity Estimates and Liana Species-area Accumulation Curves

We calculated liana and tree species richness (number of species), Fisher’s alpha, Shannon diversity index, dominance, and evenness for all independently rooted principal stems (Table S1). We compared relative abundance patterns between lianas and trees in two ways. First, we constructed frequency distributions of the log-transformed number of species falling within relative abundance classes. Second, we constructed rank-abundance curves for both lianas and trees by plotting log relative abundance of a species on the y-axis and species rank abundance on the x-axis.

We determined liana species-area curves across the entire 1000 m×500 m (50 ha) plot by calculating mean liana species richness for non-overlapping square quadrats ranging in size from 25 m^2^ (20,000 5×5 m plots) to 250,000 m^2^ (two 500×500 m plots). In total we used 13 quadrant size classes, and for each quadrat size class we calculated the mean species richness of the replicate quadrats. We sampled from the southwest corner of the plot and continued to the northeast corner to preserve the geographical integrity of liana distribution across the plot [37]. For quadrat sizes that did not divide evenly across the 50 hectares, the remaining areas that did not fit into the quadrants (the northern and eastern edges) were excluded from the analysis (methods follow [37]). We examined liana accumulation for three different minimum diameter size classes (≥1 cm, ≥5 cm, and ≥10 cm), both including and excluding clonal stems. We determined species-area curves for the three size classes for both lianas and trees to compare species accumulation with area sampled between the growth forms.

### Liana Clonality and Relative Abundance

We quantified clonal reproduction of a species as the percentage of rooted stems ≥1 cm diameter that were still physically attached to larger rooted stems in the census. We did not excavate stems to determine underground connections among stems, and clones likely decay over time, so our estimates of clonal reproduction are conservative. We calculated per-species percent clonal reproduction as the number of clones divided by the sum of the principal stems and clones for each species. We used Pearson’s correlation to examine the relationship between a species’ observed percent clonal reproduction and its log-transformed principal stem density and mean diameter – testing the hypothesis that liana species attain high abundance and size due to their ability to reproduce clonally. We excluded 12 species with very low sample sizes from this analysis (eight singletons and four species with only two individuals).

### Liana and Tree Species Spatial Distribution Patterns

We analyzed the spatial distribution patterns for the 82 liana species with more than 65 principal stems. We used 65 as our threshold abundance because this is the minimum population size to accurately detect tree habitat associations within the BCI 50-ha plot [38]. Thus, our analysis provides a consistent comparison with trees [17], [38]. For comparisons between lianas and trees, we matched each of the 82 liana species with a unique tree species in the BCI 50-ha plot having the most similar population sizes, resulting in a total of 44,971 lianas and 44,515 trees. Mean differences in population sizes between the liana and matched tree taxa were less than 4%.

To compare aggregation patterns of trees and lianas, we used Ripley’s *K* function to assess whether species distributions were significantly clumped [39], then fit a Poisson cluster model (PCM) to the species K function following the method of Plotkin et al. [40]. The PCM treats a species distribution pattern as a function of the parameters ρ, an estimate of the density of population clusters per unit area, and σ, an estimate of the mean cluster size (diameter). To determine whether the propensity of a liana species to produce clones influenced its spatial pattern across the 50-ha plot, we correlated the values of ρ and σ with the proportion of stems that were clones for the 82 most abundant lianas using Spearman’s rank correlation. We performed these tests both including and excluding clonal stems from the estimation of the PCM parameters.

To determine whether the 82 liana species differed in their within-cluster stem diameter distributions, we analyzed the spatial correlation of liana diameter distribution through the *K_mm_* function [41], using the Stoyan’s mark correlation function as a test function [42] using *R* (R development core team, 2011). We used the random labeling null model to test independence in the mark distribution [43], and the translation correction to address any bias introduced by edge effects [44]. Empirically derived values of the *K_mm_* function that were above the upper 95% quantile of the null model imply that liana stem diameters within clusters were more similar than expected by chance, suggesting that individual stems of that liana species recruited at similar times. Values of below the lower 95% quantile of the null model imply that liana stem diameters for that species were less similar than expected by chance, suggesting that the individual stems recruited sequentially over time. Values of within the 95% quantile bounds of the null model simulation indicate that there is no within-cluster liana stem diameter pattern for that species. We tested for stem diameter differences within aggregations both including and excluding clones using Wilcoxon rank sum test.

### Increasing Liana Density on BCI

To test the hypothesis that liana density has increased on BCI over the past 3 decades, we compared our data with those of Putz [1], who, in 1979, sampled all rooted liana stems in 10 randomly placed 25×40 m plots (1 ha total) on the BCI central plateau - the same general area where the BCI 50-ha plot is located. To make our sampling comparable with that of Putz [1], we computed the mean density of all rooted lianas in 500 25×40 m plots within the 50-ha plot. We compared the two datasets using a non-parametric Wilcoxon rank sum test. We compared the number of rooted stems in the ≥1 cm and ≥5 cm size classes to determine whether lianas were increasing uniformly between these two size-classes. Since most liana biomass is concentrated in stems ≥5 cm (see Results), determining the change in this size class allowed us to assess directly whether liana biomass is increasing in this forest. To examine whether lianas have increased relative to trees, we compared the change in liana density to the change in trees density (stems ≥1 cm) in the BCI 50-ha plots from 1982 until 2005 using tree data from the CTFS website.

## Results

### Liana Density, BA, and Diversity in the BCI 50-ha Plot

**Figure 1:**
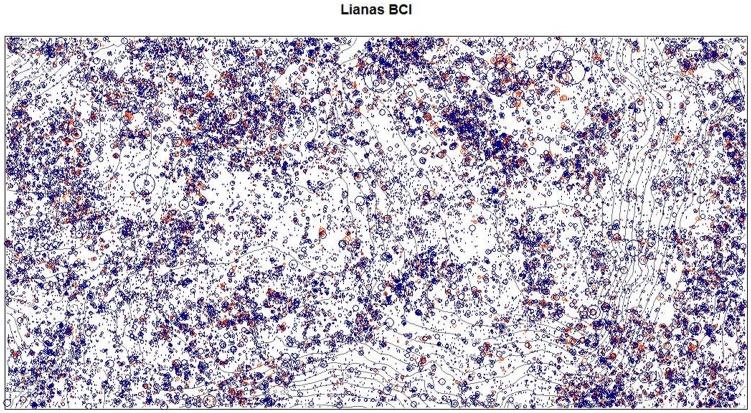
The rooted liana stems (≥1 cm diameter) in the Barro Colorado Island, Panama 50-ha plot. The plot is 1000 m on the x-axis and 500 m on the y-axis. Blue circles denote principal stems and orange circle indicate clonal stems that are rooted in the plot but still attached to a principal stem. Basal area is indicated by the size of the circle, with the largest liana 55.1 cm diameter and the smallest lianas 1 cm diameter.

**Figure 2:**
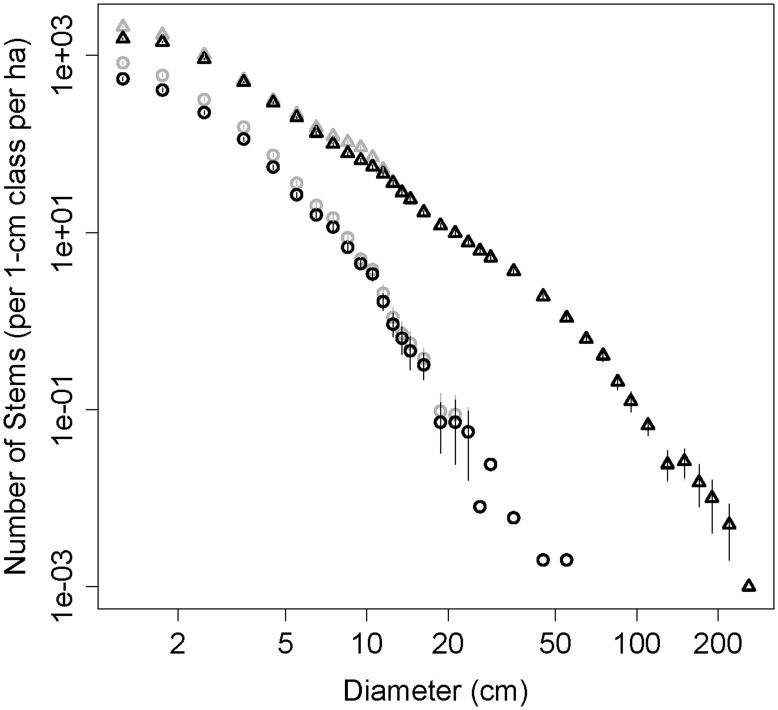
The number of liana and tree stems (log-transformed and excluding clonal stems) across 1 cm diameter size classes in the Barro Colorado Island, Panama 50-ha plot. Error bars represent 95% confidence intervals, which were calculated by bootstrapping over 20×20 m quadrants.

Over the 50-ha area there were 47,183 separately rooted liana principal stems ≥1 cm diameter (943.7 lianas ha^−1^; Figure 1). There were 428 large (≥10 cm diameter) principal stems (8.6 large lianas ha^−1^), accounting for less than 1% of all principal stems. The three largest liana stems in the BCI 50-ha plot were 55.1 cm, 42.8 cm, and 37.0 cm in diameter, and were identified as *Prionostemma asperum* (Hippocrateaceae), *Entada gigas* (Fabaceae), and *Bauhinia guianensis* (Fabaceae), respectively. The total basal area of liana principal stems ≥1 cm diameter was 36.76 m^2^ (0.74 m^2^ ha^−1^). Liana principal stem density decreased predictably with increasing diameter size classes, with the majority of all stems in the smaller size classes (Figure 2). Half (50.6%) of the total principal stem basal area (and thus more than half of the estimated biomass) was contained in stems ≥5 cm diameter and 17.3% of the principal stem basal area was contained in stems ≥10 cm diameter, even though these size classes constituted only 8.6% and 0.9% of the liana principal stems, respectively (Table 1).

**Table 1:** Size distributions of lianas and trees and shrubs for individuals ≥1 cm in the BCI 50-ha plot.

Diameter class (cm)	Lianas				Trees		
	Principal rooted stems(ha^−1^)	clonal rooted stems(ha^−1^)	Non-rooted branches(ha^−1^)	Total basal area(m^2^ ha^−1^)	Principal stems(ha^−1^)	Branches plus clones(ha^−1^)	Total basal area(m^2^ ha^−1^)
1–2	475.1	234.4	54.7	0.122	1479.2	429.2	0.308
2–5	394.7	151.6	13.1	0.417	1695.3	160.9	1.409
5–10	65.3	19.1	0.7	0.300	576.1	114.6	2.759
10–20	8.1	1.4	0.0	0.114	261.7	23.3	4.069
20–30	0.4	0.0	0.0	0.019	72.3	0.2	3.396
30–40	0.1	0.0	0.0	0.013	66.4	0.1	8.940
>50	0.0	0.0	0.0	0.000	16.7	0.0	10.811

Liana data are presented as principal rooted stems, all rooted stems (principal stems plus clones), non-rooted branches, and the sum of the basal area of all three stem types. Tree data are presented as principal rooted stems, all stems (principal stems plus clones plus branches), and the sum of the basal area of all three stem types. Lianas were censused in 2007 and the trees in 2005.

Throughout the BCI 50-ha plot, there were 162 liana species from 36 families. In comparison, in 2005 there were 299 tree species from 57 families. More than half of the liana species (83) were in the five most liana-species-rich families: Bignoniaceae (22 species), Sapindaceae (22), Fabaceae (16) Malpighiaceae (15), Apocynaceae (9). Eleven plant families had only one liana species and seven plant families had only two liana species (Table 2). The most abundant liana species was *Coccoloba excelsa* (Polygonaceae), with 9.1% of all principal stems. *Hiraea reclinata* (Malpighiaceae) and *Maripa panamensis* (Convolvulaceae) were the second and third most abundant species comprising 6.0% and 5.6% of all principal stems, respectively. The ten most abundant species comprised nearly half (48.0%) of the total principal stem density and 40.6% of the total principal stem basal area. Thirty-four species (21% of all species) were represented by fewer than 10 individuals, and 8 species (∼5% of all species) were represented by only one individual over the entire 50 ha area. Liana dominance hierarchies changed slightly at the larger sizes classes, with *Prionostemma asperum* (Hippocrateaceae) being the most abundant species at the ≥5 cm and ≥10 cm diameter size classes, and *Maripa panamensis* and *Entada gigas* (Fabaceae) being the second most abundant liana species at these larger size classes, respectively.

**Table 2:** Summary data for the lianas of the Barro Colorado Island 50-ha plot located in central Panama.

Family	Genus	Species	Stem density	Total BA (cm^2^ 50 ha^−1^)	Percent clonality	Principal stem diameter similarity within clusters	Principal stem plus clone diameter similarity within clusters
Acanthaceae	*Justicia*	*graciliflora*	9	9.81	0.00		
Acanthaceae	*Mendoncia*	*gracilis*	815	4038.07	10.80	**−**	**−**
Acanthaceae	*Mendoncia*	*retusa*	167	488.76	5.39	**0**	**0**
Apocynaceae	*Forsteronia*	*acouci*	827	3005.21	19.11	**0**	**0**
Apocynaceae	*Forsteronia*	*myriantha*	264	725.90	13.26	**+**	**+**
Apocynaceae	*Liedea*	*filisepala*	7	10.15	0.00		
Apocynaceae	*Odontadenia*	*macrantha*	30	65.39	30.00		
Apocynaceae	*Odontadenia*	*puncticulosa*	64	383.48	20.31		
Apocynaceae	*Prestonia*	*lenticellata*	20	33.20	10.00		
Apocynaceae	*Prestonia*	*mexicana*	1	1.77	0.00		
Apocynaceae	*Prestonia*	*portobellensis*	41	70.88	0.00		
Apocynaceae	*Prestonia*	*quinquangularis*	6	6.79	0.00		
Aristolochiaceae	*Aristolochia*	*cordiflora*	379	714.20	7.12	**−**	**−**
Aristolochiaceae	*Aristolochia*	*tonduzii*	14	35.80	0.00		
Asteraceae	*Heterocondylus*	*vitalbae*	1	2.01	0.00		
Asteraceae	*Mikania*	*hookeriana*	8	28.82	0.00		
Asteraceae	*Mikania*	*leiostachya*	410	1038.99	16.34	**0**	**0**
Asteraceae	*Tilesia*	*baccata*	14	39.32	7.14		
Bignoniaceae	*Amphilophium*	*paniculatum*	472	7433.52	32.20	**−**	**−**
Bignoniaceae	*Arrabidaea*	*candicans*	550	5948.63	48.18	**−**	**−**
Bignoniaceae	*Arrabidaea*	*chica*	174	1560.26	36.21	**+**	**+**
Bignoniaceae	*Arrabidaea*	*corallina*	453	3827.63	41.50	**0**	**0**
Bignoniaceae	*Arrabidaea*	*florida*	514	4015.92	33.66	**−**	**−**
Bignoniaceae	*Arrabidaea*	*patellifera*	622	5411.92	54.98	**−**	**−**
Bignoniaceae	*Arrabidaea*	*verrucosa*	1797	20241.99	55.76	**−**	**−**
Bignoniaceae	*Callichlamys*	*latifolia*	634	7281.50	29.50	**+**	**+**
Bignoniaceae	*Ceratophytum*	*tetragonolobum*	557	4175.31	31.24	**−**	**−**
Bignoniaceae	*Cydista*	*aequinoctialis*	588	7878.90	38.27	**+**	**0**
Bignoniaceae	*Cydista*	*heterophylla*	3	13.05	33.33		
Bignoniaceae	*Macfadyena*	*unguis-cati*	122	1839.27	29.51	**0**	**0**
Bignoniaceae	*Mansoa*	*kerere*	43	218.56	13.95		
Bignoniaceae	*Mansoa*	*verrucifera*	240	1081.12	42.08	**0**	**0**
Bignoniaceae	*Martinella*	*obovata*	36	286.65	25.00		
Bignoniaceae	*Paragonia*	*pyramidata*	3571	26667.75	32.32	**0**	**0**
Bignoniaceae	*Phryganocydia*	*corymbosa*	671	3158.30	13.11	**+**	**+**
Bignoniaceae	*Pithecoctenium*	*crucigerum*	63	1195.48	14.29		
Bignoniaceae	*Pleonotoma*	*variabilis*	1185	3219.52	26.16	**+**	**0**
Bignoniaceae	*Stizophyllum*	*inaquilaterum*	44	351.42	38.64		
Bignoniaceae	*Stizophyllum*	*riparium*	303	1259.49	34.98	**−**	**−**
Bignoniaceae	*Tynnanthus*	*croatianus*	20	317.50	5.00		
Boraginaceae	*Tournefortia*	*cuspidata*	4	19.68	25.00		
Boraginaceae	*Tournefortia*	*hirsutissima*	6	50.93	16.67		
Cannabaceae	*Celtis*	*iguanaea*	326	5376.42	62.88	**−**	**−**
Celastraceae	*Anthodon*	*panamense*	386	3764.83	34.20	**0**	**0**
Celastraceae	*Hippocratea*	*volubilis*	911	12312.43	30.95	**+**	**+**
Celastraceae	*Hylenaea*	*praecelsa*	149	990.65	51.01	**0**	**0**
Celastraceae	*Peritasa*	*pruinosa*	7	95.52	57.14		
Celastraceae	*Prionostemma*	*asperum*	3213	42277.70	48.02	**+**	**+**
Combretaceae	*Combretum*	*decandrum*	586	5451.43	50.34	**−**	**−**
Combretaceae	*Combretum*	*fruticosum*	26	293.38	50.00		
Combretaceae	*Combretum*	*laxum*	882	4580.05	40.59	**−**	**−**
Connaraceae	*Cnestidium*	*rufescens*	38	237.70	2.63		
Connaraceae	*Connarus*	*panamensis*	51	260.37	5.88		
Connaraceae	*Connarus*	*turczaninowii*	749	2562.89	4.01	**+**	**0**
Connaraceae	*Rourea*	*glabra*	53	313.00	5.66		
Convolvulaceae	*Bonamia*	*trichanta*	27	42.90	11.11		
Convolvulaceae	*Ipomea*	*phillomega*	2	4.31	0.00		
Convolvulaceae	*Maripa*	*panamensis*	2961	25171.71	10.37	**−**	**−**
Cucurbitaceae	*Cayaponia*	*granatensis*	177	782.04	9.60	**0**	**0**
Cucurbitaceae	*Fevillea*	*cordifolia*	49	393.43	22.45		
Cucurbitaceae	*Gurania*	*makoyana*	4	12.32	25.00		
Dilleniaceae	*Davilla*	*nitida*	659	3104.73	34.75	**+**	**0**
Dilleniaceae	*Doliocarpus*	*dentatus*	216	1686.09	29.63	**−**	**−**
Dilleniaceae	*Doliocarpus*	*major*	2065	7208.50	22.03	**−**	**−**
Dilleniaceae	*Doliocarpus*	*multiflorus*	415	4990.93	28.92	**−**	**−**
Dilleniaceae	*Doliocarpus*	*olivaceus*	2936	9145.38	33.86	**+**	**+**
Dilleniaceae	*Tetracera*	*hydrophila*	272	1654.03	35.66	**+**	**0**
Dilleniaceae	*Tetracera*	*portobellensis*	74	472.42	29.73		
Dilleniaceae	*Tetracera*	*volubilis*	11	36.23	45.45		
Dioscoreaceae	*Dioscorea*	*hondurensis*	3	3.60	0.00		
Euphorbiaceae	*Delechampia*	*dioscoreifolia*	2	10.56	0.00		
Euphorbiaceae	*Omphalea*	*diandra*	725	11376.52	48.97	**−**	**−**
Fabaceae	*Acacia*	*acanthopylla*	789	15734.28	56.27	**−**	**0**
Fabaceae	*Acacia*	*hayesii*	476	8612.64	53.15	**+**	**+**
Fabaceae	*Bauhinia*	*guianensis*	1	1075.21	0.00		
Fabaceae	*Clitoria*	*javitensis*	454	2213.73	19.82	**0**	**0**
Fabaceae	*Dioclea*	*wilsonii*	11	108.88	0.00		
Fabaceae	*Entada*	*gigas*	180	20107.56	42.78	**0**	**0**
Fabaceae	*Entada*	*polystachya*	8	696.93	16.67		
Fabaceae	*Machaerium*	*floribundum*	133	737.35	22.56	**−**	**−**
Fabaceae	*Machaerium*	*isadelphum*	9	364.64	33.33		
Fabaceae	*Machaerium*	*kegelii*	67	100.52	16.42		
Fabaceae	*Machaerium*	*microphyllum*	22	289.65	4.55		
Fabaceae	*Machaerium*	*milleflorum*	125	1948.48	43.20	**+**	**+**
Fabaceae	*Machaerium*	*pittieri*	438	3480.93	27.85	**0**	**0**
Fabaceae	*Machaerium*	*riparium*	89	1937.80	30.34		
Fabaceae	*Machaerium*	*seemanii*	203	1677.15	16.75	**+**	**+**
Fabaceae	*Rhynchosia*	*erythrinoides*	282	823.58	4.61	**0**	**−**
Gnetaceae	*Gnetum*	*leyboldii*	52	330.44	3.85		
Lamiaceae	*Aegiphila*	*cephalophora*	1464	11164.83	39.55	**+**	**0**
Lamiaceae	*Aegiphila*	*elata*	727	6224.36	23.93	**−**	**+**
Loganiaceae	*Strychnos*	*brachistantha*	127	1363.30	24.41	**0**	**0**
Loganiaceae	*Strychnos*	*darienensis*	10	29.12	0.00		
Loganiaceae	*Strychnos*	*panamensis*	262	886.75	5.73	**0**	**−**
Loganiaceae	*Strychnos*	*toxifera*	55	216.54	18.18		
Malpighiaceae	*Adelphia*	*hiraea*	1422	7683.91	15.89	**−**	**−**
Malpighiaceae	*Bronwenia*	*wurdackii*	28	396.09	60.71		
Malpighiaceae	*Heteropteris*	*laurifolia*	227	1910.26	30.84	**0**	**0**
Malpighiaceae	*Hiraea*	*fagifolia*	145	998.80	29.66	**0**	**0**
Malpighiaceae	*Hiraea*	*faginea*	75	495.43	28.00		
Malpighiaceae	*Hiraea*	*grandifolia*	876	4433.83	20.78	**0**	**−**
Malpighiaceae	*Hiraea*	*reclinata*	354	2957.93	28.81	**+**	**+**
Malpighiaceae	*Hiraea*	*smilacina*	3473	16871.07	18.37	**0**	**−**
Malpighiaceae	*Mascagnia*	*divaricata*	175	3204.84	12.00		
Malpighiaceae	*Mascagnia*	*ovatifolia*	115	1871.38	7.83	**+**	**+**
Malpighiaceae	*Stigmaphyllon*	*lindenianum*	389	1814.05	13.62	**+**	**+**
Malpighiaceae	*Tetrapteris*	*discolor*	215	1237.16	12.56	**0**	**0**
Malpighiaceae	*Tetrapteris*	*goudotiana*	36	236.88	8.33		
Malpighiaceae	*Tetrapteris*	*seemannii*	24	163.77	12.50		
Malpighiaceae	*Tetrapteris*	*tinifolia*	19	128.48	15.79		
Malvaceae	*Byttneria*	*aculeata*	59	305.62	30.51		
Melastomataceae	*Adelobotrys*	*adscendens*	4	11.66	0.00		
Menispermaceae	*Abuta*	*racemosa*	600	4889.36	15.50	**0**	**0**
Menispermaceae	*Chondodendron*	*tomentosum*	81	608.94	8.64	**0**	**0**
Nyctaginaceae	*Pisonia*	*aculeata*	116	2416.09	32.76	**0**	**−**
Palmae	*Desmoncus*	*orthacanthos*	4	6.50	25.00		
Passifloriaceae	*Passiflora*	*ambigua*	18	69.30	16.67		
Passifloriaceae	*Passiflora*	*auriculata*	128	309.41	7.81	**0**	**0**
Passifloriaceae	*Passiflora*	*nitida*	5	30.39	0.00		
Passifloriaceae	*Passiflora*	*vitifolia*	44	80.74	4.55		
Phytolaccaceae	*Trichostigma*	*octandrum*	5	105.83	40.00		
Piperaceae	*Piper*	*multiplinervium*	226	1093.12	44.69	**+**	**+**
Polygalaceae	*Securidaca*	*diversifolia*	9	77.43	33.33		
Polygonaceae	*Coccoloba*	*excelsa*	7976	27840.21	46.33	**−**	**−**
Rhamnaceae	*Gouania*	*colombiana*	266	1117.36	32.71	**−**	**−**
Rubiaceae	*Chiococca*	*alba*	13	182.96	30.77		
Rubiaceae	*Chomelia*	*psilocarpa*	75	668.74	54.67		
Rubiaceae	*Uncaria*	*tomentosa*	206	8983.56	53.88	**0**	**0**
Sapindaceae	*Paullinia*	*baileyi*	530	825.76	4.15	**0**	**0**
Sapindaceae	*Paullinia*	*bracteosa*	504	1028.05	9.52	**+**	**0**
Sapindaceae	*Paullinia*	*fibrigera*	1216	7443.32	26.73	**+**	**+**
Sapindaceae	*Paullinia*	*fuscescens*	61	383.81	32.79		
Sapindaceae	*Paullinia*	*glabrata*	57	481.28	19.30		
Sapindaceae	*Paullinia*	*glomerulosa*	10	9.94	0.00		
Sapindaceae	*Paullinia*	*mallophylla*	115	345.21	6.96		
Sapindaceae	*Paullinia*	*pinnata*	2	3.55	0.00		
Sapindaceae	*Paullinia*	*pterocarpa*	454	908.63	4.19	**0**	**0**
Sapindaceae	*Paullinia*	*rugosa*	400	1352.07	16.75	**0**	**0**
Sapindaceae	*Paullinia*	*turbacensis*	365	623.99	1.92	**0**	**0**
Sapindaceae	*Serjania*	*atrolineata*	7	56.06	85.71		
Sapindaceae	*Serjania*	*circumvallata*	264	1680.83	11.74	**−**	**−**
Sapindaceae	*Serjania*	*cornigera*	1	1.77	0.00		
Sapindaceae	*Serjania*	*membranacea*	15	119.82	40.00		
Sapindaceae	*Serjania*	*mexicana*	847	2661.30	43.80	**−**	**−**
Sapindaceae	*Serjania*	*paucidentata*	1	24.63	0.00		
Sapindaceae	*Serjania*	*pluvialiflorens*	41	249.71	21.95	**0**	**0**
Sapindaceae	*Serjania*	*pyramidata*	55	289.95	29.09		
Sapindaceae	*Serjania*	*rhombea*	12	21.65	0.00		
Sapindaceae	*Serjania*	*trachygona*	77	113.78	11.69	**0**	**0**
Sapindaceae	*Thinouia*	*myriantha*	1398	18046.93	36.55	**+**	**0**
Smilacaceae	*Smilax*	*domingensis*	10	15.74	50.00		
Smilacaceae	*Smilax*	*febrifuga*	71	116.29	26.76		
Smilacaceae	*Smilax*	*spinosa*	17	40.97	17.65		
Smilacaceae	*Smilax*	*spissa*	1	0.79	0.00		
Smilacaceae	*Smilax*	*spp.*	27	252.47	3.70		
Solanaceae	*Solanum*	*aturense*	53	247.73	15.09		
Solanaceae	*Solanum*	*lanceifolium*	46	185.33	10.87		
Trigoniaceae	*Trigonia*	*rugosa*	6	28.73	16.67		
Urticaceae	*Urera*	*lianoides*	95	263.10	23.16	**0**	**0**
Verbenaceae	*Petrea*	*volubilis*	2614	9424.56	11.74	**+**	**+**
Vitaceae	*Cissus*	*erosa*	26	589.21	3.85		
Vitaceae	*Cissus*	*microcarpa*	67	802.99	13.43		
Vitaceae	*Vitis*	*tiliifolia*	49	618.07	38.78		
	*Unidentified*		817	6706.20	15.69		

For each of the 162 species, we list the density of all rooted stems (principal stems plus clones), the total liana basal area (principal stems plus clones plus branches) the percentage of observed clonal stems, and diameter similarity within clusters of principal stems and all rooted stems (principal stems plus clones). Analyses of diameter similarity within clusters were conducted on the 82 most common species; a plus sign means that the rooted stems in the same cluster were more similar in diameter than expected by chance, a minus sign means that diameters varied more within clusters than expected by chance, and “0” means that diameter variation was not significantly different from what was expected by chance.

Synonymous species names: *Adelphia hiraea = Mascagnia hiraea, Aristolochia cordiflora = Aristolochia gigantea, Aristolochia tonduzii = Aristolochia chapmaniana, Bauhinia guianensis = Bauhinia excisa, Bronwenia wurdackii = Banisteriopsis cornifolia, Clitoria javitensis = Clitoria portobellensis, Coccoloba excelsa = Coccoloba parimensis, Davilla nitida = Davilla multiflora, Desmoncus orthacanthos = Desmoncus isthmius, Dioscorea hondurensis = Dioscorea Haenkeana, Doliocarpus major = Doliocarpus punctatus, Doliocarpus multiflorus = Doliocarpus guianensis, Entada polystachia = Entadopsis polystachya, Forsteronia viridescens = Forsteronia acouci, Gurania makoyana = Gurania seemanniana, Hiraea smilacina = Hiraea quapara, Hippocratea volubilis = Hippocratea versicolor, Justicia graciliflora = Beloperone graciliflora, Machaerium kegelii = Machaerium pachyphyllum, Machaerium seemannii = Machaerium campylocarpum, Mansoa kerere = Pachyptera kerere, Mascagnia ovatifolia = Mascagnia nervosa, Mendoncia litoralis = Mendoncia retusa, Odontadenia macrantha = Odontadenia grandiflora, Paullinia turbacensis = Paullinia wetmorei, Petrea volubilis = Petrea aspera, Pithecoctenium crucigerum = Pithecoctenium echinatum, Prestonia mexicana = Prestonia allenii, Prestonia quiquangularis = Prestonia acutifolia, Rhynchosia erythrinoides = Rhynchosia pyramidalis, Serjania pyramidata = Serjania decapleuria, Smilax febrigua = Smilax panamensis, Smilax domingensis = Smilax lanceolata, Solanum aturense = Solanum siparunoides, Solanum lanceifolium = Solanum lanciifolium, Tetrapterys goudontiana = Tetrapterys macrocarpa, Tilesia baccata = Wulffia baccata.*

**Figure 3:**
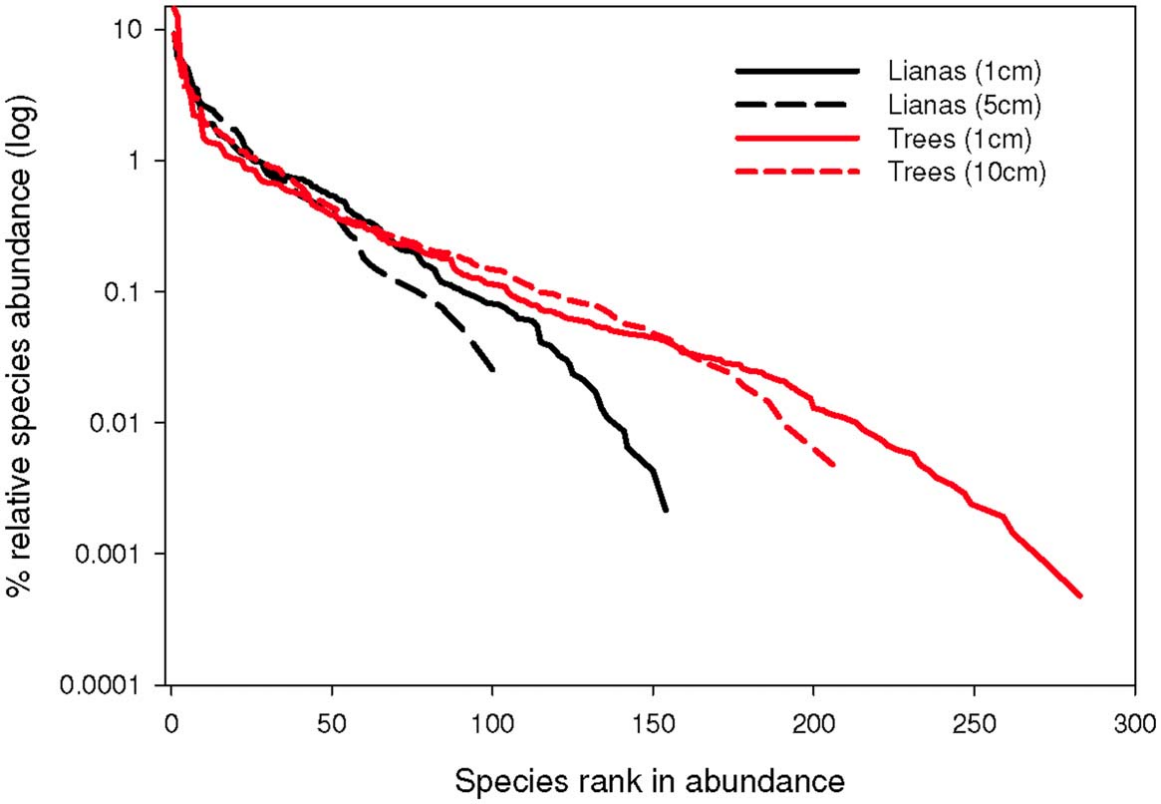
Liana and tree rank-abundance curves over a 50 ha area on Barro Colorado Island, Panama.

In the context of all woody plants (lianas and trees ≥1 cm), liana principal stems constituted 18.5% of total woody plant density (Figure 2) and 35.3% of the species in the BCI 50-ha plot, but only 2.2% of the total woody plant basal area (Table 3). Compared to trees, liana stems were smaller and their frequency decreased faster with increasing diameter (Figure 2). Liana diversity, as measured by the Shannon diversity index, was slightly higher than trees because the lower species richness of lianas was balanced by higher evenness (Table 3). Indeed, liana rank-abundance curves had lower y-intercepts, were initially shallower, and were subsequently steeper than those of trees (Figure 3).

**Table 3:** Comparison of lianas and freestanding woody plants (trees and shrubs) in their total abundance and community structure for individuals ≥1 cm in the BCI 50-ha plot.

Diversity Index	Rooted lianaindividuals	All rooted lianas	Rooted trees & Shrubs
Stem Density	47,185 (18.5%)	67,449 (24.5%)	208,387
Total Basal Area (m^2^)	36.76 (2.2%)	49.22 (2.9%)	1672.09
Species Richness	162 (35.1%)	162 (35.1%)	299
Fisher’s Alpha	21.19		34.32
Shannon Diversity	4.01		3.96
Dominance	0.031		0.049
Evenness	0.339		0.175

Liana data are presented as both rooted principal stems only (individuals excluding clones) and all rooted stems (principal stems plus clones). Lianas were censused in 2007 and the trees in 2005. The percentage of total woody species are listed in parentheses.

### Clonal Reproduction

Clonal reproduction of lianas was surprisingly high; there were 20,264 clonal stems (≥1 cm diameter) rooted in the 50-ha plot and still connected to a principal stem. Adding clonal stems increased liana density 43% to 67,447 total lianas rooted in the 50-ha area (1350 lianas ha^−1^; Figure 1). Clonal stems added 12.46 m^2^ in basal area, which increased liana basal area 34% to 49.21 m^2^ over the 50 hectares (0.98 m^2^ ha^−1^), and liana clones were prevalent even at very large size classes (Figure 2). Rooted liana density and basal area (including clones) as a percentage of all woody individuals were 24.5% and 2.9% of all woody stems. Species varied from zero to 63% in the percentage of all stems that were clonally derived, and only 3 of the 129 species with more than 10 individuals lacked rooted clones. There was a weak but significantly positive correlation between species’ observed percent clonal reproduction and the relative density of principal stems (p = 0.03, R^2^ = 0.03, n = 149). There was a strong significant positive relationship between species’ observed percent clonal reproduction and its mean stem size throughout the plot (p<0.0001, R^2^ = 0.37, n = 149).

### Liana Species-area Patterns

**Figure 4:**
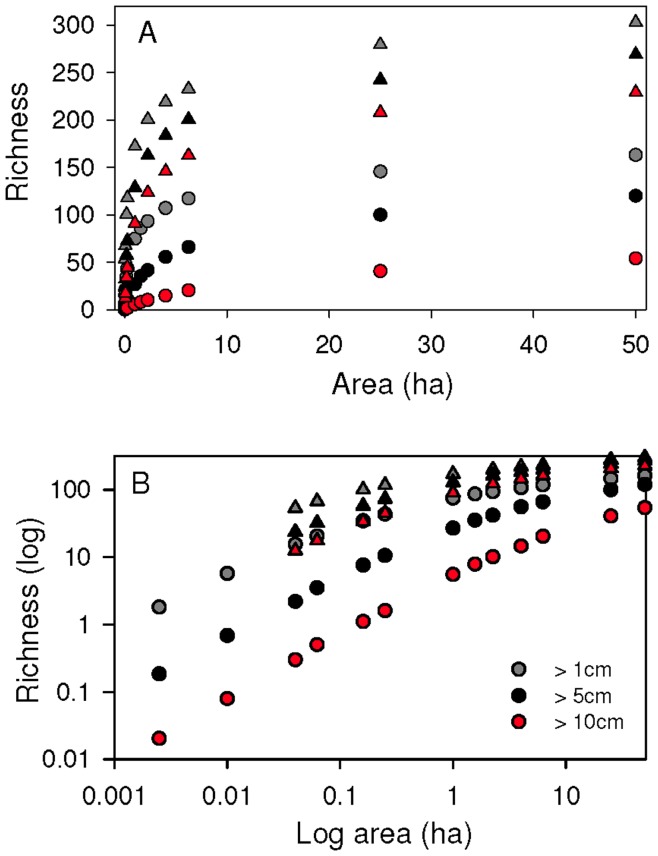
Liana and tree species-area curves for three size classes over 50 ha on Barro Colorado Island, Panama. Triangles represent trees and circles represent lianas. Panel A is based on untransformed data and panel B is based on transformed data.

Across the 50-ha plot, mean liana richness was 75 species ha^−1^ based on 100×100 m plots. Liana species richness increased rapidly to approximately 125 species at 6 ha, and then increased more gradually up to 50 ha (Figure 4). Approximately seventy percent of the species were included in the first six hectares; however, species were still accumulating at the maximum plot size, suggesting that rare species would continue to accumulate if we sampled more than 50 hectares. Nonetheless, our census of the BCI 50-ha plot captured over 90% of liana species reported for the entire 1600 ha island [33]. The pattern of liana species accumulation was similar for all three size-classes; however, only around one-third of the species present in the 50-ha plot reached the large liana size-class (≥10 cm diameter). Species accumulation curves followed similar trajectories for trees and lianas (Figure 4).

### Liana Spatial Distribution Across the BCI 50-ha Plot

**Figure 5:**
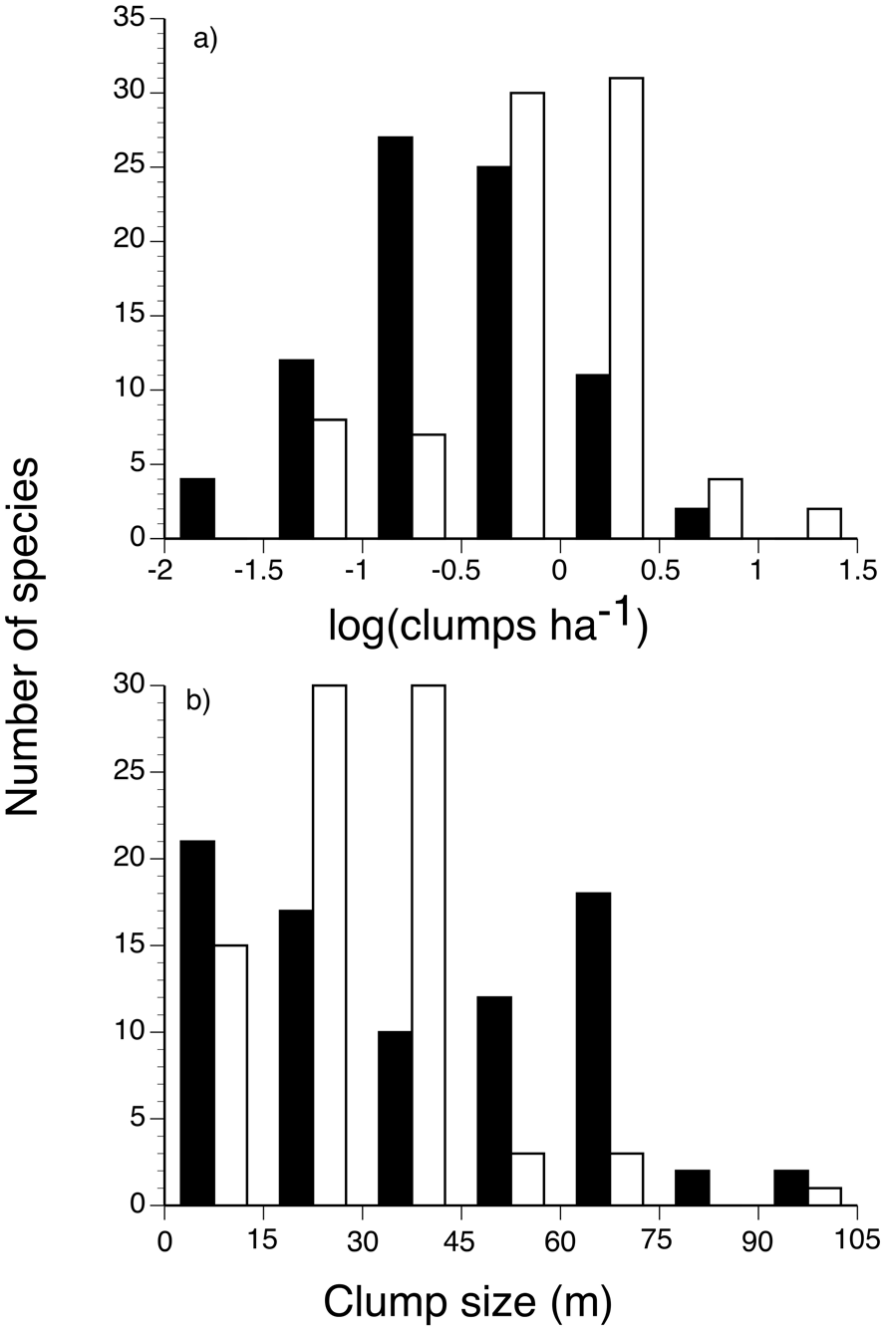
Spatial clustering parameters for 82 liana species with >65 apparent genets (filled bars), and for a sample of 82 tree species with similar population sizes (open bars) in the Barro Colorado Island, Panama 50-ha plot. Parameters are: a) ρ, the density of individuals within clusters; and b) σ, the mean cluster size.

Distributions of the 82 most abundant liana species (excluding clones) were significantly spatially aggregated at the 50 ha scale. When the population aggregation pattern was further decomposed into cluster density (clusters per ha) and size (mean cluster area), we found that lianas had significantly fewer population clusters than the matched sample of tree species (Figure 5a; Wilcoxon test, W = 1911, p<0.001; median cluster densities: lianas 0.30 ha^−1^, trees 0.83 ha^−1^), indicating that lianas had more individuals per cluster. In contrast, liana and tree cluster size did not differ (Figure 5b; Wilcoxon test, W = 3490, p = 0.48), although variance in cluster size was greater for lianas than trees (K-S test, D = 0.27, p<0.01). Including liana clones did not significantly change our results for the density of clusters in the plot (Wilcoxon test, W = 3306, p>0.05) or cluster size (Wilcoxon test, W = 3112, p>0.05); however, including liana clones increased the number of lianas within clusters.

To explore the effects of clonal reproduction on liana aggregation patterns, we examined the relationship between the proportion of clonal stems per species and the values of ρ and σ for the 82 most abundant lianas. We found that species with more clonally derived stems had fewer clusters (Spearman r = −0.28, p = 0.012) and, when clones were excluded from the analysis, species with greater clonality also had smaller mean cluster size (Spearman r = −0.27, p = 0.016). Thus, clonal reproduction resulted in fewer but more densely packed liana aggregation patterns.

Similar stem sizes within clusters could indicate that the individuals of that species recruited at the same time, while a significant variation in stem sizes could indicate that individuals of that species colonized sequentially over time. We found that 24 of the 82 species (29%) had more similar stem diameter sizes per cluster than expected by chance, whereas 25 species (30%) had significantly more variation in stem diameters within clusters than expected by chance (Table 2). The remaining 40% of the species did not have a significant correlation among stem sizes within clusters (p>0.05). Including clonal stems did not substantively change these findings, and only one species (*Aegiphila elata*) showed a complete switch in stem size similarity when the clonal stems were included in the analysis (Table 2). The different stem diameter organization within clusters (more or less variation than expected by chance) among species was not correlated with the number of individuals of a species nor the percentage of clonal reproduction (p>0.05).

### Increasing Liana Density on BCI

From 1979 until 2007, liana density increased by 75% for stems ≥1 cm diameter and by nearly 140% for stems ≥5 cm diameter. We found 134.9 (±3.7 se) rooted liana stems ≥1 cm per 25×40 m plot in 2007, significantly more than the 77.3 (±10.8) recorded by Putz in 1979 [1] (P = 0.008). For lianas ≥5 cm, we found 10.2 (±0.3) liana stems per 25×40 m plot versus 4.3 (±1.3) in 1979 (P<0.001). Thus, lianas have increased an average of 21 stems ≥1 cm ha^−1^ year^−1^ and 2.1 stems ≥5 cm ha^−1^ year^−1^, and compounding increases of 2.0% and 2.7% per year, respectively (assuming arithmetically constant rate of increase). In contrast, the density of trees ≥1 cm on the BCI 50-ha plot decreased 11.5% from 235,338 individuals 50 ha^−1^ in 1982 to 208,387 in 2005, an average decrease of 23 trees ha^−1^ year^−1^, or 0.6% per year. Tree density peaked in 1990 at 244,059 individuals 50 ha^−1^ and then decreased more than 14.5% over the subsequent 15 years.

## Discussion

Our study represents the largest and most comprehensive assessment of tropical liana density, diversity, and distribution to date. Lianas in the BCI 50-ha plot are both abundant and diverse, and they are increasing in density and biomass, possibly due to global change [27], [30]. Lianas are spatially clumped throughout the BCI 50-ha plot – more so than trees – which may be due to the ability of lianas to rapidly colonize treefall gaps, particularly via clonal reproduction [1], [3], [17], [19]. Furthermore, lianas had higher community evenness and a lower proportion of rare species than did the tree community. By studying how liana density, BA, and species richness change across large spatial scales, and how these patterns differ from those of trees, we gain insight into where and when lianas will have the largest effect on trees, as well as the processes governing community assembly of woody tropical plants.

### BCI Liana Community Compared to Other Forests

In the BCI 50-ha plot there were a total of 67,447 rooted lianas (1350 ha^−1^ including rooted clones; 944 ha^−1^ excluding clones) and 162 species, constituting 24.5% of the woody stems and 35.3% of the woody species (75 species ha^−1^). In relation to other forests, liana density and species richness on BCI was moderate. For example, liana density and diversity was much higher at Yasuní National Park in Ecuador, where there were around 1600 rooted lianas (≥1 cm diameter) and approximately 180 species in an area totaling one hectare subsampled throughout a 30 ha area [45]. If the accumulation in liana individuals and species with area at Yasuní is similar to that of BCI, we would expect approximately 80,000 individuals and 390 species in the Yasuní 50-ha plot. Tree diversity is also high at Yasuni (>1,100 tree species in the 50-ha plot), and thus lianas composed around 25% of the woody species at Yasuni. In a highly seasonal forest in lowland Bolivian Amazon, liana density was exceedingly high, with 2471 lianas ha^−1^ (≥2 cm diameter), yet was less species-rich than BCI (51 species ha^−1^; [11]). In contrast, mean liana density on BCI was 21% higher (including only principal stems) than in the ever-wet forest of La Selva Biological Station in Costa Rica, which had 777 lianas (≥1 cm diameter) ha^−1^ and 60 species in 0.78 ha [20]. The relatively moderate liana density and basal area on BCI is consistent with global multi-forest comparisons showing that liana density and basal area [26], [46], as well as species richness [47], tend to increase with increasing seasonality and decreasing mean annual rainfall.

### Clonal Reproduction

Clonal reproduction was common in the liana community of BCI. Adding clonal stems to principal stems increased rooted liana density and BA by 43% and 34%, respectively. Our estimate of clonal reproduction is conservative because connections among liana ramets may be underground or may decay after the ramet becomes established, e.g. [1], [5], [48]. Thus, we likely considered many lianas to be apparent genets when they were actually clones that had lost their attachment to the parent stem or whose attachment to the parent stem was not visible.

The frequency of clonal reproduction was highly variable among liana species. Both common and rare liana species displayed evidence of varying levels of clonal reproduction, with some common species exhibiting extremely high levels of clonal reproduction (>50% of stems being clonal) and other common species exhibiting very low levels of clonal reproduction (<5% of stems being clonal). The significant positive correlation between observed clonal reproduction and species principal stem density suggests that clonal reproduction is generally an advantageous strategy for lianas; however, the very low R^2^ indicates that it not a strong predictor of stem density for a given species. In contrast, the strong positive correlation between clonal reproduction and mean stem diameter indicates that stems of species that produced many clones tended to be larger than species that produced few clones. This latter relationship may be driven by rapid growth of clonal stems, which could gain an advantage from resources supplied by the maternal stem or, alternatively, larger stems may simply have more resources to produce clonal stems.

The degree of clonal reproduction can influence liana population structure. For liana species with a high propensity for clonal reproduction, all apparently distinct individuals could theoretically belong to a single giant clone. Although few studies have examined liana population structure over large distances using genetic techniques, e.g. [49], observational studies have shown that liana stems can extend long distances from their root systems by growing laterally from tree crown to tree crown throughout the forest, extending more than 500 m from their initial rooting point in extreme cases [1]. When a tree falls, the lianas in that tree’s crown are commonly pulled into the gap, where they resprout vigorously, create new root systems [1], [3], [50], and eventually climb back up to the forest canopy far from the principal rooting point [5], [19]. Repeated cycles of lianas climbing to the forest canopy, growing laterally away from their root systems, falling to the forest floor, and then resprouting and growing back to the forest canopy may allow genetically identical liana stems to spread slowly through the forest over large spatial and temporal scales.

### Liana Distribution and Spatial Structure within the 50-ha Plot

Lianas were spatially clumped in the BCI 50-ha plot - a pattern that could be driven by the strong colonization and regeneration responses of lianas to common forest disturbances, such as the formation of treefall gaps. Because lianas respond rapidly to disturbance [1], [3], [50], [51], treefall gaps may be the foci of liana recruitment [8], [9], and this type of small-scale disturbance may explain the clumped distribution of lianas throughout the forest. The clumped distribution pattern may be strongly influenced by highly clonal liana species (Table 1), which had much higher stem density per cluster and may be more disturbance adapted than less clonal species [52]. Consequently, the distribution of liana species within a forest may be driven largely by treefall gaps [3], [8], [26], [53], and liana species with a high frequency of clonal reproduction may be responsible for the pattern of densely packed liana aggregations throughout the forest. Furthermore, by killing canopy trees [10], lianas create the very niche that promotes their regeneration, which may largely explain how liana diversity is maintained in the forest.

Contrasting patterns of stem diameter size among liana species within aggregations may also be related to disturbance and may indicate different liana colonization strategies. We hypothesize that the presence of many similar-sized individuals within aggregations indicate a one-step colonization strategy, in which most stems of a liana species, including clones that were subsequently separated from the parent stem, recruit at one time, presumably in the high light environment of a treefall gap [3]. In contrast, liana species with a wide variation in stems sizes within aggregations colonize via a consecutive-step strategy, in which individuals recruit over a longer time span. This latter pattern may be indicative of more shade-tolerant species that recruit under a closed canopy.

Determining differences in colonization strategies among liana species may provide insight into the mechanisms driving liana increases in neotropical forests [27]. For example, if liana increases are driven by increasing disturbance, we would expect more disturbance-adapted and highly clonal liana species to increase in abundance. In contrast, if other mechanisms such as increasing aridity, nitrogen deposition, hunting, or elevated atmospheric CO_2_ are responsible for liana increases [27], [30], we may expect that both disturbance-adapted and highly shade-tolerant liana species will increase in abundance. If, as we hypothesize, the variation in stem size within aggregations is a good indicator of shade-tolerance, then species-specific patterns of liana stem-size distribution throughout the forest and within aggregations provide useful data for testing hypotheses to explain the observed increases in liana abundance and biomass in tropical forests.

### Increasing Liana Density on BCI

By comparing our data to those collected nearly 30 years earlier, we found that liana density on BCI increased 75% for stems ≥1 cm diameter and nearly 140% for stems ≥5 cm diameter – a finding that is consistent with other metrics of increasing lianas on BCI. For example, the amount of liana leaf litter compared to that of trees on the BCI 50-ha plot increased 40% from 1986 until 2002 [28]. Liana flower productivity also increased faster than that of trees during this same period [29]. The percentage of trees that carried (and presumably competed with) lianas increased from 32% in 1967–1968 to 75% in 2007, and the number of trees with severe liana infestation in their crowns (>75% crown coverage by lianas) increased 65% from 1996 to 2007 [10]. Because trees with severe liana infestation have twice the probability of mortality of trees with lesser or no liana infestation [10], liana increases will result in decreased tree growth and survival, which will lead to decreased forest-wide carbon storage [16], [30]. Our data, combined with previous studies, confirm that lianas are increasing on BCI, and thus the effects of lianas are also likely to be increasing at this site.

Liana increases are not unique to BCI. Over the past three decades, lianas have increased dramatically relative to trees throughout the neotropics, and there are now more than ten published studies to support this emerging pattern [27]. In addition to Panama, lianas have been reported to increase in forests in Bolivia [54], Brazil [55], Costa Rica [19], [56], French Guiana [22], and in subtropical forests in South Carolina, USA [57]. The relative increase in liana density and biomass represents an important structural and compositional change in neotropical forests.

Lianas may be increasing on BCI and in other neotropical forests due to elevated rates of disturbance [58], which would contribute to an increasingly aggregated pattern of lianas. Lianas respond rapidly to disturbance with high rates of colonization and growth [1], [3], [8], [50], [51], and thus disturbance is currently one of the leading explanations for liana increases [27]. Lianas may also be increasing due to increasing aridity and the occurrence of El Niño droughts, e.g. [56], [59], [60]. Lianas peak in abundance in highly seasonal forests [26], [46] and on drier soils within the BCI forest [17]. The driver of this pattern may be the ability of lianas to grow during seasonal droughts when competing trees grow far less [26], [62], [63]. This dry season growth advantage hypothesis [26] is consistent with the increase in the proportion of drought-tolerant trees on BCI over the past 25-years [64]; see also [56]. Liana increases could also be explained by higher nitrogen deposition and atmospheric CO_2_ [30], [65], and the combination of increasing aridity, disturbance, nitrogen deposition and atmospheric CO_2_ all may operate synergistically to favor lianas over trees [27], [30]. Future research that combines characterization of liana life history strategies with data on changes in individual species abundances and spatial patterns could help distinguish among these putative hypotheses to explain the increase in lianas on BCI and in other neotropical forests.

## Supporting Information

Table S1 **Equations used to calculate Fisher’s alpha, Shannon Index, Dominance, and evenness.**
(DOC)
