# Supplementary material for: Liana Abundance, Diversity, and Distribution on Barro Colorado Island, Panama
Source: PLoS One. 2012 Dec 21;7(12):e52114. doi: 10.1371/journal.pone.0052114 (PMC3528767; doi:10.1371/journal.pone.0052114)
Supplement: Table S1 — Equations used to calculate Fisher’s alpha, Shannon Index, Dominance, and evenness. (DOC) [file pone.0052114.s001.doc]

**Fisher’s alpha:**

$$s=\alpha\ln\left(\frac{n}{\alpha}\right)$$

*s* = number of species

*n* = number of individuals

**Shannon index:**

*n*_i_ is the number of individuals of species i

**Dominance:**

**Evenness:**

$\frac{H}{\ln(n)}$
